# Steatosis is a Lot More than Holes in Hepatocytes

**DOI:** 10.4103/1319-3767.45045

**Published:** 2009-01

**Authors:** Subrata Chakrabarti

**Affiliations:** Department of Pathology, Schulich School of Medicine, The University of Western Ontario, and London Health Sciences Centre, London, Ontario, Canada

Chronic hepatitis C infection remains a common and significant cause of liver disease leading to end-stage cirrhosis and liver failure. It is also a common indication for the necessity of a liver transplant. Histologically, chronic inflammation and fibrosis are characteristic features of this disease. The stage and grade of the disease process is measured by the amount of fibrosis and severity of the inflammation. Focal fatty change is a known histological finding of hepatitis C infection. However, the exact significance of such a finding is not clear.

In this issue, Zubair *et al*.[1] demonstrated that the presence of fat, especially macrovesicular steatosis, is associated with a higher stage of fibrosis. They used a routine histological analysis as well as a morphometric evaluation to demonstrate an association. The authors suggested that such fat may result from mitochondrial oxidative stress. Hence, along with viral infection and inflammation, the presence of fat may also modulate the progression of this disease.

Fat accumulation is known to cause fibrosis in alcoholic and non alcoholic fatty liver disease. Such changes may occur due to the release of adipocytokines causing tissue damage.[2–4] In addition, mitochondrial oxidative stress may lead to the activation of proinflammatory and fibrogenic factors.[5–7] It is, however, interesting to note that only macrovesicular steatosis has a pronounced effect on an advanced hepatitis C induced lesion. This point is of significant interest from a pathogenetic point of view. However, although this study was not designed to answer the reasons behind such findings, it suggested a possible relationship. In previous studies, it has been shown that serum HCV RNA levels correlate with histological liver damage and concur with steatosis and a high viral load acts together with steatosis in accelerating the progression of liver injury.[8] In an earlier study, this group had also demonstrated that patients with genotype 3a showed a significantly higher prevalence of steatosis compared with those infected with other genotypes.[9] In another study, although hepatic steatosis was not associated with HCV RNA levels, it was associated with being overweight, hepatic fibrosis, and triglyceride levels in chronic hepatitis C infection.[10]

Nevertheless, an inevitable question that may arise is whether an assessment of a change in fat is to be included in a routine pathology report to determine a prognosis. This is not easy to answer. However, larger multi-center studies may be required to address such questions. A large-scale study with a detailed clinico-pathological correlation may provide valuable insight. Furthermore, investigations as to the specific mechanisms by which only macrovesicular fat causes tissue damage are very important. Such analysis may have far reaching implications and may also be of importance in other liver diseases associated with fat accumulation in the hepatocytes.

Understanding pathogenetic mechanisms is fascinating but a difficult undertaking. Extensive basic science research is needed to address these questions. We need to have concerted efforts involving hepatologists, pathologists, translational researchers, and basic scientists to identify such pathogenetic mechanisms since only a better understanding of such pathogentic mechanisms will lead to the development of specific treatment for advanced tissue damage in hepatitis.
